# Neurosurgery for severe refractory OCD in Malta – clinical audit of a new service

**DOI:** 10.1192/j.eurpsy.2026.11618

**Published:** 2026-07-31

**Authors:** R. Galea, M. Caruana, L. Meilak, M. Krueger, A. Grech, L. Zrinzo

**Affiliations:** 1 National Mental Health Services, Attard; 2 Mater Dei Hospital, Msida, Malta; 3 University College London Hospitals NHS Foundation Trust, London, United Kingdom

## Abstract

**Introduction:**

The prevelance of OCD is approximately 2-3%. Its impact depends on its severity and effect on functionality. Multiple aetiological factors are recognised, including: genetic, neurophysiological, and socio-environmental contributors. Success rate of non-surgical management; psychopharmacotherapy and psychotherapy, ranges between 40-60%, aiming at a 40-50% symptom decrease. Stereotactic neurosurgery provides a 60-70% success rate in treatment resistant OCD, with low risk of complications.

**Objectives:**

This paper reports a prospective cohort study of individuals experiencing treatment resistant OCD in Malta who underwent Stereotactic Anterior Capsulotomy (SACAPS).

**Methods:**

Individuals experiencing treatment resistant OCD are referred to the neuropsychiatry team; exhausting conventional treatment modalities. Six participants underwent SACAPS after clearance by the Mental Health Commissioner as part of safeguarding legislation in Malta. Pre- and post-operative psychometric testing was performed. We report the change in the Yale Brown Obsessive-Compulsive Scale (Y-BOCS), the Hamilton Depression Rating Scale (HAM-D) as well as a qualitative narrative from the participants’ lived experiences. Necessary authorisations were gathered while data was solely collected from participants’ notes.

**Results:**

A total of six individuals underwent SACAPS for OCD between 2017-2025. Response is defined as ≥35% reduction on the Y-BOCS. A reduction of 20-35% is a partial response. Mean (SD) Y-BOCS scores improved from 32.5 (6.1) to 20.5 (10.4). Three (50%) participants showed full response on the Y-BOCS with a mean improvement of 60%, including one patient achieving full remission (YBOCS ≤8). One patient (16.66%) showed a partial response of 30% change, and two (33.33%) were “non-responders”. One of the non-respondent participants experienced an improved quality of life and decreased burden of symptoms reflected in the time spent admitted in a psychiatric hospital; decreasing from 191/591 (32%) to 37/2913 days (1%) until time of write-up. Complex refractory cases require multiple treatment and augmentation strategies elevating the risk of side-effects impacting the quality of life. No post-operative complications were identified in our cohort. These results echo international statistics on neurosurgical interventions for treatment resistant OCD. Qualitative data substantiated these benefits and improved quality of life reflected in decreased distress and improved function.

**Conclusions:**

Around 40% of patients experiencing OCD have poor response to non-surgical treatment. This statistical picture confirms that neurosurgical interventions in treatment refractory OCD in well-selected patients can provide potential significant improvements with limited complications or residual side-effects. Further observation of the current sample, larger samples, and improved referral systems would support further Maltese patients with severe refractory OCD.

**Disclosure of Interest:**

None Declared

